# Aberrant MFN2 transcription facilitates homocysteine‐induced VSMCs proliferation via the increased binding of c‐Myc to DNMT1 in atherosclerosis

**DOI:** 10.1111/jcmm.14341

**Published:** 2019-05-18

**Authors:** Long Xu, Hongyi Hao, Yinju Hao, Guo Wei, Guizhong Li, Pengjun Ma, Lingbo Xu, Ning Ding, Shengchao Ma, Alex F. Chen, Yideng Jiang

**Affiliations:** ^1^ Ningxia Vascular Injury and Repair Research Key Laboratory Ningxia Medical University Yinchuan China; ^2^ School of Basic Medical Sciences Ningxia Medical University Yinchuan, Ningxia China; ^3^ The People's Hospital in Ningxia Hui Autonomous Region Yinchuan China; ^4^ Department of Clinical Medicine Ningxia Medical University Yinchuan, Ningxia China; ^5^ Department of Surgery University of Pittsburgh School of Medicine Pittsburgh Pennsylvania

**Keywords:** c‐Myc, DNA methylation, homocysteine, mitofusin‐2, proliferation

## Abstract

It is well‐established that homocysteine (Hcy) is an independent risk factor for atherosclerosis. Hcy can promote vascular smooth muscle cell (VSMC) proliferation, it plays a key role in neointimal formation and thus contribute to arteriosclerosis. However, the molecular mechanism on VSMCs proliferation underlying atherosclerosis is not well elucidated. Mitofusin‐2 (MFN2) is an important transmembrane GTPase in the mitochondrial outer membrane and it can block cells in the G0/G1 stage of the cell cycle. To investigate the contribution of aberrant MFN2 transcription in Hcy‐induced VSMCs proliferation and the underlying mechanisms. Cell cycle analysis revealed a decreased proportion of VSMCs in G0/G1 and an increased proportion in S phase in atherosclerotic plaque of APOE^−/−^ mice with hyperhomocystinaemia (HHcy) as well as in VSMCs exposed to Hcy in vitro. The DNA methylation level of MFN2 promoter was obviously increased in VSMCs treated with Hcy, leading to suppressed promoter activity and low expression of MFN2. In addition, we found that the expression of c‐Myc was increased in atherosclerotic plaque and VSMCs treated with Hcy. Further study showed that c‐Myc indirectly regulates MFN2 expression is duo to the binding of c‐Myc to DNMT1 promoter up‐regulates DNMT1 expression leading to DNA hypermethylation of MFN2 promoter, thereby inhibits MFN2 expression in VSMCs treated with Hcy. In conclusion, our study demonstrated that Hcy‐induced hypermethylation of MFN2 promoter inhibits the transcription of MFN2, leading to VSMCs proliferation in plaque formation, and the increased binding of c‐Myc to DNMT1 promoter is a new and relevant molecular mechanism.

## 1 INTRODUCTION

Atherosclerosis is a chronic progressive disease which is characterized by the formation of atheromatous plaque in the intimal layer that mainly derived from the deregulation of cell behaviour, such as the activation of macrophages and abnormal vascular smooth muscle cells (VSMCs) proliferation and so on.^1^, ^2^ It has been indicated that homocysteine (Hcy) is an independent risk factor for atherosclerosis and it can induce endothelial dysfunction, foam formation and promote VSMC proliferation.^3^, ^4^ As an important component of the medial layer of blood vessels, VSMCs migration and proliferation with subsequent formation of intimal thickening is important for the development of atherosclerotic lesions. However, little is known about the underlying mechanisms regarding the aberrant VSMCs proliferation during atherosclerotic plaque formation.

Mitochondrial GTPase mitofusin‐2 (MFN2), also known as a hyperplasia suppressor gene, is widely distributed in mammalian.^5^ Recently, it was reported that MFN2 plays an essential role in mitochondrial fusion, which regulates mitochondrial morphology and function in multiple cell types.^6^ Meanwhile, MFN2 has been demonstrated to be low expressed in various types of human malignant tumours, such as gastric cancer, hepatocellular cancer, colorectal cancer and breast cancer.^7^, ^8^ Apart from its role in cancer, clinical evidence revealed that dysfunction of MFN2 is also involved in the pathophysiology of several cardiovascular diseases including hypertension, restenosis after angioplasty, cardiac hypertrophy and cardiac oxidative stress injury.^9^, ^10^ Intriguingly, MFN2 was found to exert an anti‐proliferative effect by inducing more cells at the G0/G1 phase in cultured MCF‐7 cells and a rat carotid artery balloon‐injury model.^11^ These data led us to suggest that destabilization of MFN2 may play a role in the excessive proliferation of VSMCs during the formation of atherosclerotic plaque.

Hcy is a product of the methionine cycle, it is involved in one‐carbon methyl group‐transmethylation pathway and acts as a methyl donor when it is converted to S‐adenosyl‐methionine (SAM).^12^ Accumulating evidences demonstrated that aberrant DNA methylation induced by Hcy is associated with various diseases including atherosclerosis, osteoporosis, uraemia and alcoholism.^13^, ^14^ DNA methylation could directly modulate gene transcription via recruiting chromatin remodelling proteins and modulating the binding affinities of specific transcription factors.^15^ In eukaryotes, it is well accepted that transcriptional activity of the specific genes was influenced by epigenetic marks and interplay between transcription factors and the cis‐elements of specific promoters in time and space, which is closely related to gene expression.^16^ MFN2 expression has been reported to be regulated by a series of transcription factors that control mitochondrial biogenesis and functions, including ERRalpha and MEF2. As a highly conserved transcription factor, c‐Myc could drive cell stress, proliferation and apoptosis through integrating multiple cellular signals and mediating a transcriptional response.^17^ Previous studies demonstrated that c‐Myc is involved in the transcriptional regulation of the specific genes in different ways, and one of its regulatory functions involved gene transcription driven by the binding to E‐box sequences located on the gene promoter regions.^18^ In addition to its function as an activator, c‐Myc also can repress transcription of genes through interaction with epigenetic modification such as DNA methylation. c‐Myc may orchestrate DNMTs to regulate common target genes linked to multiple networks in the development and progression of diseases. In lung cancer cells, DNMT3b could be recruited to the promoter region of RAS association domain family1A (RASSF1A) by c‐Myc to silence its expression through DNA hypermethylation.^19^ A comprehensive understanding of this dynamic interplay will set the stage, not only for the design of novel treatment strategies, but also for the discovery of pan‐cellular transcription factor regulatory strategies to predict disease risk, therapy response and patient prognosis^20^ and the dynamics of the mode of binding to DNA has changed this postulate and paved the way for new therapies targeted against VSMCs proliferation.

In this study, we aimed to elucidate the role of MFN2 in Hcy‐induced VSMCs proliferation during the formation of atherosclerotic plaque and the relevant molecular mechanisms. Our findings revealed that c‐Myc binding to DNMT1 promoter positively regulates DNMT1 expression, and DNMT1‐mediated DNA hypermethylation of MFN2 promoter thereby inhibits MFN2 expression in VSMCs proliferation induced by Hcy. These findings shed new insight into the mechanism of Hcy‐induced VSMCs proliferation in atherosclerosis and may be a therapeutic tool in the treatment of Hcy‐induced cardiovascular diseases.

## 2 MATERIALS AND METHODS

### 2.1 Chemicals and reagents

Cell‐Light EdU Apollo 567 In Vitro Imaging Kit was from RiboBio (Guangzhou, China), DL‐Homocysteic Acid (DL‐Hcy), 5‐aza‐2′‐deoxycytidine (5‐AZC), 10058‐F4, anti‐*β*‐actin antibody were from Sigma (St. Louis, USA), antibodies against MFN2, DNMT1, c‐Myc, PCNA, p27, Ki‐67 were from Abcam (Cambridge, UK), HRP conjugated goat anti‐rabbit IgG and goat anti‐mouse‐IgG were from Jackson ImmunoResearch (West Grove, USA), cell cycle detection kit was from Bestbio (Shanghai, China).

### 2.2 Animals

Six‐week‐old male APOE^−/−^ mice with C57BL/6J genetic background were provided by Animal Center of Peking University (Beijing, China). They were housed in a temperature‐controlled (24°C) facility with a 12 hours light/dark cycle. After 1 week of acclimatization, APOE^−/− ^mice were randomly divided into two groups (*n* = 6 each) and fed with regular diet (APOE^−/−^+NC) or fed with regular diet plus 1.7% methionine (APOE^−/−^+HMD). After 15‐week experimental diets, mice were killed with pentobarbital (50 mg/kg body weight), and aortic tissues were frozen in liquid nitrogen and stored at 80°C until further analysis. All animal experiments were approved by the Ethics of Animal Experiments of the Health Science Center of Ningxia Medical University.

### 2.3 Haematoxylin and eosin (HE) and oil red O staining

The aortas in APOE^‐/‐^mice were flushed with saline and embedded in OCT after sacrifice. Frozen sections were cut in 4 μm thickness, followed by staining with haematoxylin and eosin (HE) and Oil Red O staining. Details about the staining can be found in our previous study,^3^ and lipid‐stained lesions were measured by digitizing morphometry and reported in mm^2^ per lesion.

### 2.4 Detection of serum Hcy

Blood samples collected from the mice were centrifugated at 3000 *g* for 10 min at 4°C after standing at room temperature for 30 min, then serum concentrations of Hcy were measured by automatic biochemistry analyzer (SIEMENS, Germany).

### 2.5 Cell culture

Human VSMCs were cultured in Dulbecco's modified Eagle's medium (DMEM) supplemented with 7% FBS, 100 μg/mL streptomycin and 100 IU/mL penicillin. Cells at 80% confluence were subsequently treated with Hcy at the concentrations of 0 (control), 50, 100, 200 and 500 μmol/L for 72 hours, medium were changed every 12 hours due to the short half‐life of Hcy. Recombinant adenoviruses expressing DNMT1 or c‐Myc gene were purchased from HANBIO (Shanghai, China), the plasmid expressing MFN2, siRNAs specifically targeting MFN2, DNMT1, c‐Myc and control siRNA were synthesized by Gene Pharma (Shanghai, China), and they were transfected into cells according to the manufacturer's protocol.

### 2.6 EdU proliferation assay

VSMCs proliferation was evaluated using Cell‐Light EdU Apollo 567 in vitro Imaging Kit according to the manufacture's instruction. Cells in confocal dish were treated as above and incubated with EdU solution for 2 hours. Then they were fixed with 4% paraformaldehyde for 20 minutes, and treated with 0.5% Triton‐X‐100 for another 20 minutes at room temperature. After washing with PBS, cells were incubated with 1× Apollo® reaction cocktail for 30 minutes. Subsequently, nuclei were counterstained with Hoechst 33342 stain solution for 30 minutes at room temperature. Images were captured by OLMPUS FV3000 confocal laser scanning microscope (Tokyo, Japan), and the proliferation rate of cells was assessed with the proportion of EdU‐positive nucleus (red) to blue fluorescent nucleus by counting six microscopic fields randomly in each well in three separate experiments.

### 2.7 Immunofluorescent staining

The cold acetone of aortas root in APOE^−/− ^mice were fixed with for 30 minutes, permeabilized with 0.2% Triton X‐100 for 8 minutes, blocked with PTS (1% goat serum in PT) at 4°C and then incubated with primary antibodies (PCNA, p27, Ki‐67, α‐SMA, c‐Myc and DNMT1) respectively overnight at 4°C. Subsequently, the specimens were incubated with corresponding TRITC‐ or FITC‐conjugated secondary antibody at 37°C for 1 hour, nuclei were stained with DAPI for 5 minutes at room temperature. Digital images were acquired with OLMPUS FV3000 confocal laser scanning microscope (Tokyo, Japan).

### 2.8 Cell cycle analysis

VSMCs treated with different concentrations of Hcy were trypsinized and washed with cold PBS for three times, and then fixed in 75% ethyl alcohol at 4°C overnight. After washing with PBS, cells were incubated with 1 mg/mL RNase A at 37°C for 30 minutes, then stained with PI for 1 hour in the dark. Cell cycle was analysed in a FACS Calibur flow cytometer (BD Biosciences, CA). The percentages of cells in the G1, S and G2‐M phases were determined by analysis using Modfit LT software (BD, Topsham, ME).

### 2.9 qRT‐PCR

Total RNA was isolated from cells with RNeasy Mini Kit (Qiagen, Germany) according to the manufacturer's protocol, and the cDNA was synthesized by the Revert Aid first strand cDNA synthesis kit (Fermentas, USA). Primer sequences of PCNA, p27, MFN2, DNMT1, c‐Myc and GAPDH were listed in Table 1. qRT‐PCR was performed using a miScript SYBR Green PCR Kit (DBI® Bioscience, Germany). The expression levels of mRNA were normalized using GAPDH as a reference gene. All experiments were done in triplicate.

**Table 1:** Primer sequences for qRT‐PCR analysis

Gene	Primer sequence (5′→3′)	Length (bp)	Annealing temperature
PCNA	Forward: 5′‐GAAGTTGGAGGCACTCAAGG‐3′	145	61.5°C
	Reverse: 5′‐GCAGCGGTAGGTGTCGAAGCC‐3′_		
p27	Forward: 5′‐GCAAGTACGAGTGGCAAGAGGTG‐3′	118	62.0°C
	Reverse: 5′‐CCGCTGACATCCTGGCTCTCC‐3′		
MFN2	Forward: 5′‐GGTGCTCAACGCCAGGATTCAG‐3′	197	61.5°C
	Reverse:5′‐GTCGAACCGCCTCTGCAATCTG‐3′		
DNMT1	Forward: 5′‐ATCGAGACCACGGTTCCTCCTTC‐3′	101	61°C
	Reverse: 5′‐TAACTCTCCTGCTCCACCAC‐3′		
c‐Myc	Forward: 5′‐CGAGGAGAATGTCAAGAGGCGAAC‐3′	174	60.5°C
	Reverse: 5′‐GCTTGGACGGACAGGATGTATGC‐3′		
GAPDH	Forward:5′‐AGAAGGCTGGGGCTCATTTG‐3′	258	58°C
	Reverse:5′‐AGGGGCCATCCACAGTCTTC‐3′		

### 2.10 Western blot

Whole cell lysates were prepared as described previously.^15^ A total of 30 μg protein were separated by 8% SDS‐PAGE, and then electro‐transferred onto PVDF membrane (Millipore, USA). Membranes were blocked with 5% non‐fat milk in PBST and incubated with indicated antibody at 4°C overnight. After washing with PBST for three times, membranes were incubated with horseradish peroxidase (HRP)‐conjugated secondary antibodies for 2 hours at room temperature. Protein bands were visualized using ECL solution after PBST washing, and the relative expression of each target protein was measured using *β*‐actin as the reference.

### 2.11 MassArray methylation analysis

Genomic DNA was purified from cultured cells using a genomic DNA isolation kit (Thermo Scientific, USA) followed by bisulfite conversion of DNA (1 μg) with EpiTect bisulfite kit (Qiagen, German). Sequence MassARRAY platform (CapitalBio, Beijing, China), which was composed of matrix‐assisted laser desorption/ ionization time‐of‐flight (MALDI‐TOF) mass spectrometry and combined with RNA base‐specific cleavage was used to analyse MFN2 methylation quantitatively (Gen‐Bank Accession Number: NM_001127660.1). The amplified region was close to the transcription start site, and PCR primers were listed as following: forward primer 5′‐aggaagagagGTAGTTTTAGGAGAGGGAGAGGAGA‐3′ and reverse primer 5′‐cagtaatacgactcactat agggagaaggctCACCACTACACTCCAACTTAAATAAAA‐3′. Methylation standards (0%, 20%, 40%, 60%, 80% and 100% methylated genomic DNA) were used for data normalization.

### 2.12 ChIP qPCR assay

ChIP assay was performed using a ChIP assay kit (Millipore, USA) according to the manufacturer's instruction. Briefly, the specimens were cross‐linked with 1% formaldehyde at 37°C for 8 minutes, and then terminated with 0.125 mol/L glycine. After washing with ice cold PBS for three times, the specimens were resuspended in ChIP lysis buffer containing 1% SDS, 10 mmol/L EDTA, 50 mmol/L Tris and protease inhibitor. Anti‐c‐Myc antibody was used to precipitate the DNA‐protein complex. Input and IgG was used for normalization. Primers used in ChIP assay: Forward 1: GCGTGGTGGCTCACACCTGTA; Reverse 1: GATTACAGGCGTGCACCA CCACCAT; Forward 2: TAGCGCATCTCCGTTTGCACTGG; Reverse 2: GTAATGCGGCAGACACTACTA GGAA. Amplified DNA bands were separated by 4% agarose gels, and images were obtained using a gel imaging system (Bio‐Rad Laboratories, CA).

### 2.13 Dual‐Luciferase reporter assay

Dual‐Luciferase reporter assays were performed using Luciferase Reporter Gene Assay Kits (Promega, USA) according to the manufacturer's instructions. Briefly, 200 ng of a pGL3 reporter containing targeted regions and 5 ng of Renilla Luciferase vector (pRL‐TK; Promega, USA) were co‐transfected into HEK293 cells using Lipofectamine 2000 (Invitrogen, USA). After 48 hours, the luciferase activity was determined using Dual‐Glo Luciferase Assay System (Promega, USA). The data were combined to calculate differences in firefly luciferase activity normalized by Renilla Luciferase activity.

### 2.14 Co‐immunoprecipitation (Co‐IP) assay

Cells were lysed in a lysis buffer containing protease inhibitor on ice. After centrifugation, the supernatant was incubated with an anti‐DNMT1, anti‐c‐Myc or normal rabbit IgG respectively at 4°C overnight followed by incubation with Dynabeads Protein G. Immune complex was separated by SDS‐PAGE and proceeded for Western blot analysis.

### 2.15 Statistical analysis

Results are expressed as the mean ± SD from at least three independent experiments. The data were analysed using one‐way ANOVA and additional analysis using the Student Newman‐Keuls test for multiple comparisons within treatment groups or *t*‐test for two groups. *P* < 0.05 was considered to be statistically significant.

## 3 RESULTS

### 3.1 Hcy promotes VSMCs proliferation in atherosclerotic plaque formation

**Figure 1:**
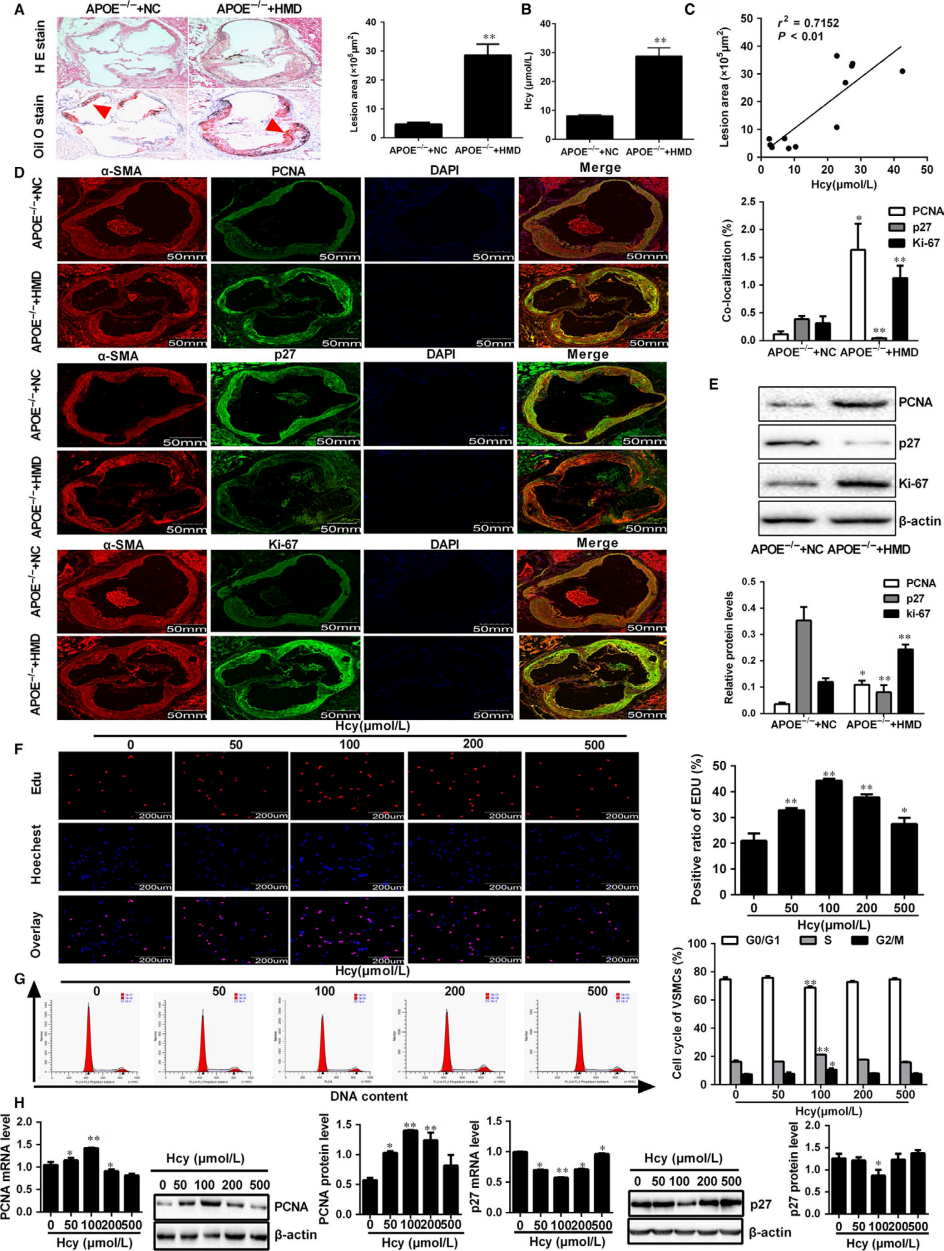
VSMCs proliferation induced by Hcy was involved in atherosclerotic plaque formation. A, Representative images and quantification of atherosclerotic plaque at the aortic roots were observed by Haematoxylin and eosin (H&E) and Oil Red O staining. Scale bar, 500 μm. The red arrow indicated a plaque region. B, The serum Hcy levels in APOE^−/−^ mice were determined by automated biochemical analyzer. C, The correlation of the atherosclerotic plaque area with Hcy levels was evaluated by Pearson correlation analysis. D, Representative immunofluorescence images and quantification of p27, PCNA and Ki‐67 (green) co‐localization with α‐smooth muscle actin (α‐SMA, red) in APOE^−/−^ mice. Nuclei were stained with DAPI (blue). Yellow indicated presence of both red and green staining, scale bar, 50 mm. E, Western blot was used for quantitative analysis the protein expression of PCNA, p27 and Ki‐67 in aorta of APOE^−/− ^HMD and APOE^‐−/− ^NC mice. F, DNA synthesis of VSMCs were detected by EDU incorporation assays following treatment with different concentrations of Hcy, scale bar, 200 μm. Red and blue fluorescence represent EdU‐positive nuclei and general nuclei, respectively (magnification, ×400). The percentage of EdU‐positive nuclei to total nuclei was quantified. G, DNA content of VSMCs were stained with propidium iodide and analysed by flow cytometry after treatment with different concentrations of Hcy (0, 50, 100, 200 and 500 μmol/L). H, The expressions of PCNA and p27 were detected by Western blot and qRT‐PCR analyses in VSMCs exposed to different concentrations of Hcy. The experiment was performed in triplicate, and the representative images are shown. *P < 0.05, **P < 0.01, compared to APOE^−/−^+NC or control group

Plaques formation is a major event in atherosclerosis related activation of macrophages and proliferation of VSMCs.^1^, ^2^ To gain a better understanding of the aberrant VSMCs proliferation in atherosclerotic plaque formation, we established atherosclerosis model as described previously,^21^ which was characterized by a markedly increased atherosclerotic lesions in aortic root and serum Hcy levels in APOE^−/− ^mice fed with high‐methionine diet as well as a significant positive correlation between serum Hcy levels and atherosclerotic lesions (Figure 1A‐C). VSMCs proliferation is a complex and stepwise process, which is regulated by various cell cycle proteins including p27, PCNA and Ki‐67. Among them, p27 could suppress cell cycle progress through arresting cells in the G0/G1 phase, while PCNA and Ki‐67 was closely related to DNA synthesis and cell mitosis respectively.^22^ We next conducted double immunofluorescent staining with antibodies against (PCNA, Ki‐67 and p27) and α‐smooth muscle actin (α‐SMA), a marker for smooth muscle cells. As shown in Figure 1D, Hcy induced a substantial increase in the number of PCNA and Ki‐67 puncta, which also co‐localized well with α‐SMA‐positive cells. Conversely, an opposite effect was observed on p27, suggesting that VSMCs proliferation induced by Hcy might dependent on G0/G1 arrest. Moreover, Western blot was used to analyse the VSMC proliferation marker in the mice aorta and we found that the protein expression of PCNA and Ki‐67 were obviously enhanced in aorta of APOE^−/−^HMD mice compared with APOE^−/−^NC mice, while the protein level of p27 apparently reduced (Figure 1E). To further support our observation, VSMCs were incubated with different concentrations of Hcy and subjected to EdU incorporation assays, the results showed that incorporation of VSMCs was accelerated by Hcy and the significant effects was observed at the concentration of 100 μmol/L (Figure 1F). In addition, the proliferation of VSMCs was assessed by flow cytometry analysis. The results showed that Hcy could result in the transition from G0/G1 to the S phase in VSMCs, particular at the concentration of 100 μmol/L (Figure 1G). In agreement with the results above, the expression of PCNA was also significantly elevated in response to Hcy, and the expression of p27 was suppressed (Figure 1H). Collectively, these results suggested that Hcy enhances VSMCs proliferation via promoting G1/S transition in the atherosclerotic plaque formation.

### 3.2 MFN2 attenuated VSMCs proliferation in the atherosclerotic plaque formation

**Figure 2:**
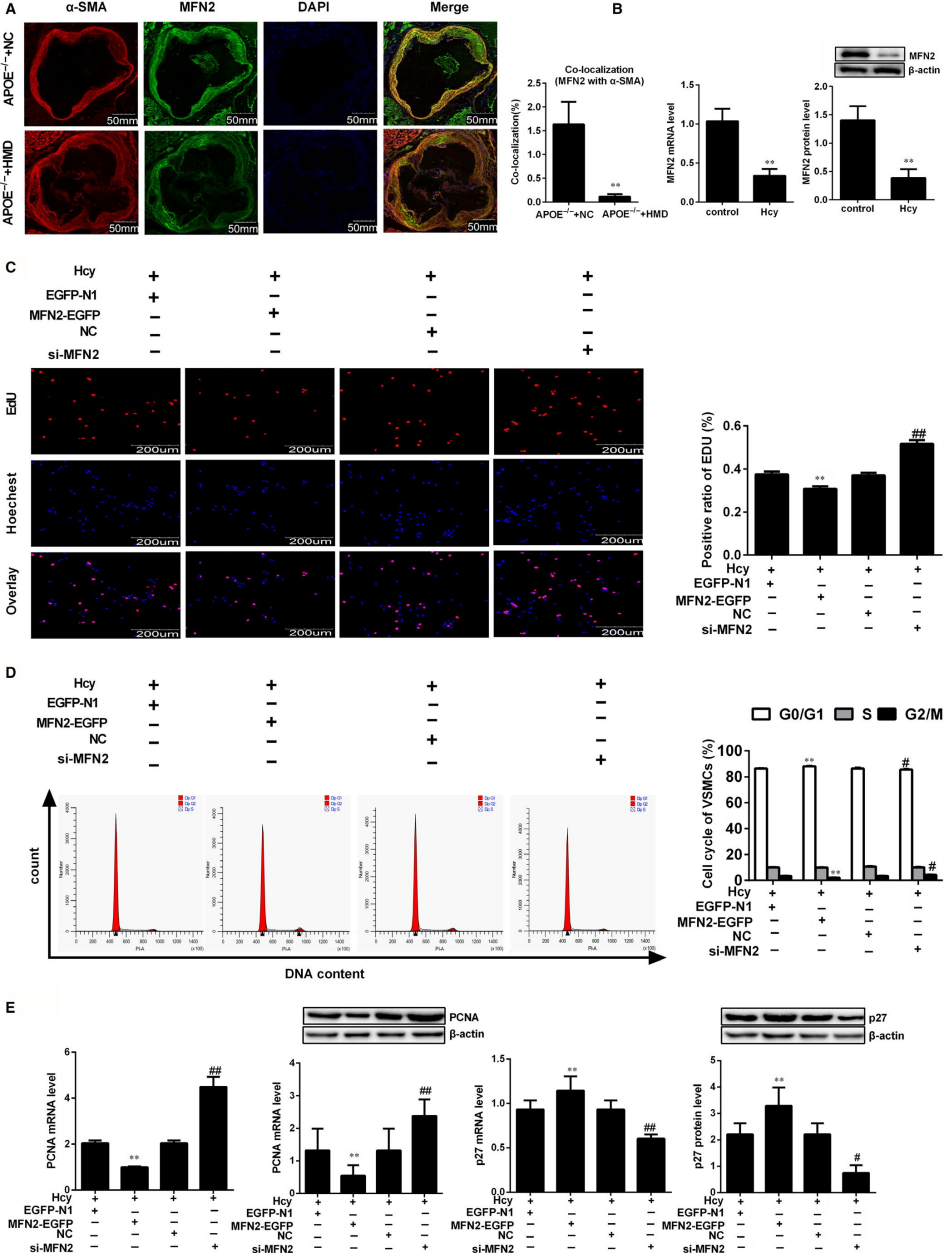
Down‐regulation of MFN2 is required for the VSMCs proliferation induced by Hcy. A, Representative immunofluorescence images of MFN2 (green) co‐localized with α‐SMA (red) in APOE^−/−^ mice. Nuclei were stained with DAPI (blue). Yellow indicated presence of both red and green staining, scale bar, 50 mm. The average MFN2 intensity and co‐location with α‐SMA was quantified, respectively. B, MFN2 expression were detected by qRT‐PCR and Western blot in VSMCs treated with 100 μmol/L Hcy. C, EdU incorporation assay was performed to evaluate DNA synthesis after VSMCs transfected with EGFP‐MFN2 and MFN2 siRNA respectively, scale bar, 200 μm. D, Flow cytometry analysis was performed to examine the cell‐cycle distribution of VSMCs after transfected with MFN2‐EGFP and MFN2 siRNA. E, qRT‐PCR and Western blot were used to detect the expression of PCNA and p27 in VSMCs transfected with MFN2‐EGFP and MFN2 siRNA. The experiment was performed in triplicate, and the representative images are shown. **P < 0.01, compared to APOE^−/−^+NC group or Hcy+EGFP‐N1 group, #*P* < 0.05, ##*P* < 0.01, compared to Hcy+NC group

MFN2 is involved in a set of biological processes, including apoptosis and proliferation, and it was reported that overexpression of MFN2 results in a cell‐cycle arrest at G0/G1 phase.^11^ To determine whether MFN2 participates in VSMCs proliferation during atherosclerotic plaque formation, double immunofluorescent staining using antibodies against MFN2 and α‐SMA was conducted in APOE^−/−^ mice. As shown in Figure 2A, MFN2 was co‐localized with α‐SMA in aortic root, and double positive cells for α‐SMA and MFN2 were notably decreased in APOE^−/−^ mice fed with high‐methionine diet. Likewise, the mRNA and protein expression of MFN2 were evidently decreased in VSMCs treated with Hcy, compared to control group (Figure 2B). Subsequently, MFN2‐EGFP and MFN2 siRNA were transfected into VSMCs respectively along with confirming the success and efficiency of them (Supplementary Figure S1A,B). Further experiments showed that overexpression of MFN2 in VSMCs decreased the percentage of EdU‐positive cells by EdU assay in the presence of Hcy, and silencing MFN2 with specific siRNA obtained the opposite effect (Figure 2C), suggesting that the down‐regulation of MFN2 expression following Hcy may be involved in VSMCs proliferation during the atherosclerotic plaque formation. Based on the aforementioned results above, cell cycle analysis showed that overexpression of MFN2 results in a significant increase in G0/G1 phase cells, which is accompanied by a synchronous decrease in the percentage of S‐phase cells with the treatment of Hcy, and knockdown of MFN2 obtained the contrary results, indicating that cell cycle arrest at G0/G1 phase is responsible for the anti‐proliferation effects of MFN2 in Hcy‐treated VSMCs (Figure 2D). Meanwhile, the expression of PCNA and p27 were detected in MFN2 overexpressed VSMCs and the results showed that overexpression of MFN2 could decrease the expression of PCNA and increase the expression of p27, while knock‐down of MFN2 gave the opposite results (Supplementary Figure S1C), implying that MFN2 inhibited proliferation of VSMCs. Similar results were also observed in VSMCs treated with Hcy (Figure 2E), meaning that MFN2 suppressed the Hcy‐induced proliferation of VSMCs. Collectively, these results demonstrated that MFN2 results in a cell‐cycle arrest at G0/G1 phase, and VSMCs proliferation induced by Hcy in atherosclerotic plaque formation is at least partly through the inhibition of MFN2 expression.

### 3.3 Hcy inhibited MFN2 transcriptional activity via DNMT1 leading to VSMCs proliferation

**Figure 3:**
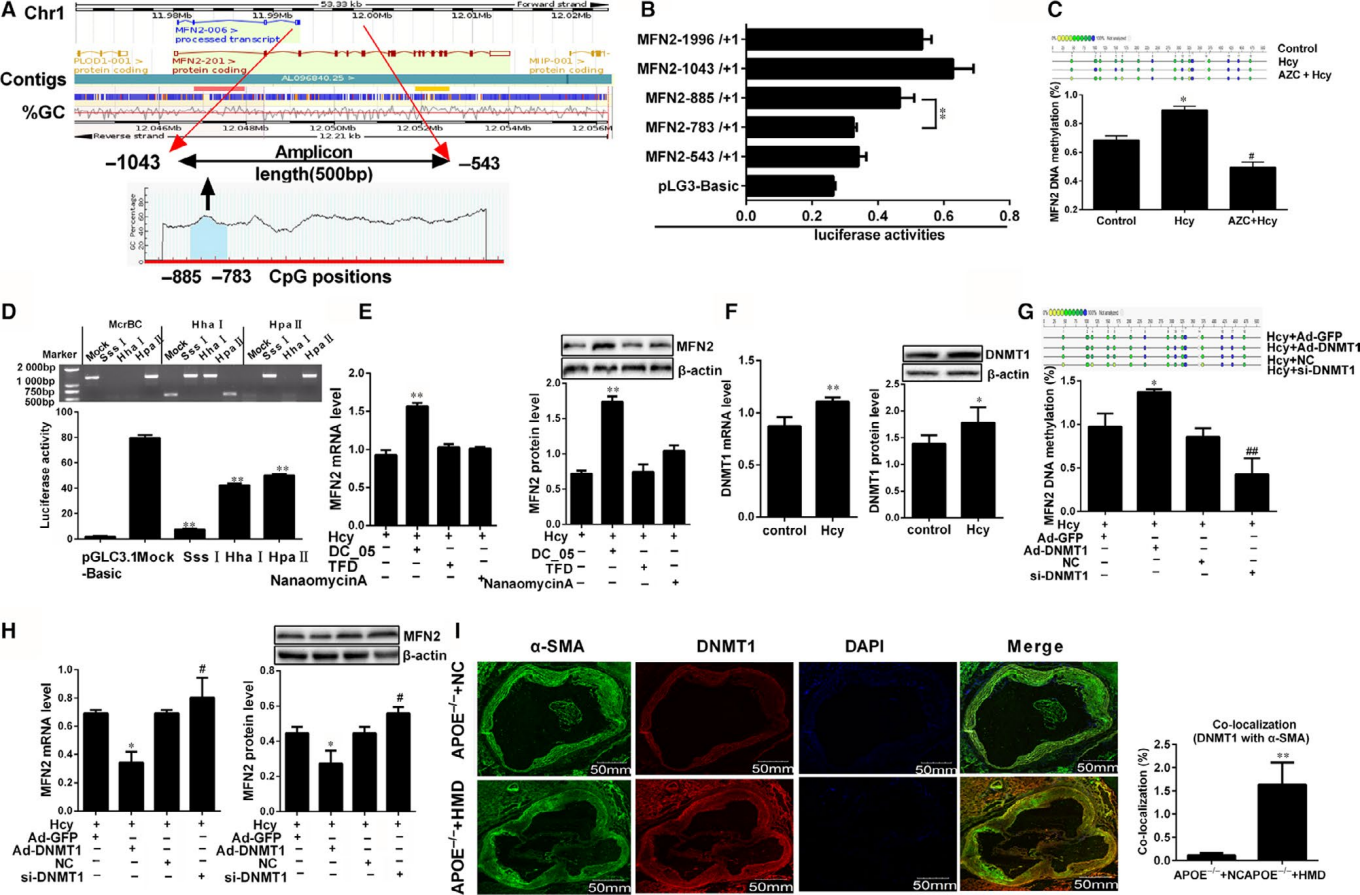
Hcy inhibited MFN2 transcriptional activities via DNMT1 in VSMCs. A, Analysis of MFN2 gene location, amplicon size and CpG islands in the amplicon. Methylation profile of CpG sites for MFN2 gene. B, The luciferase assay of activities of MFN2 promoter region (−1996/+1, −1043/+1, −885/+1, −783/+1 and −543/+1). **P < 0.01, compared to MFN (−783/+1) group. C, MassARRAY Quantitative Methylation was performed to determine methylation status at the MFN2 promoter in VSMCs. Gray circles indicate not analysed methylation values owing to CpGs with high‐ or low‐mass Dalton peaks falling outside the conservative window of reliable detection for the EpiTYPER software, the colour of the circles represents the per cent of methylation in each CpG site. D, The proximal promoter region (−1403 to +1) of MFN2 was digested with McrBC, XhoI, HhaI, HpaI and HpaII to confirm the methylation status, then Dual‐Luciferase reporter assay was performed to ascertain the activity of the MFN2 proximal promoter differentially methylated with SssI, XhoI, HhaI, HpaI and HpaII methylase in VSMCs transfected with luciferase reporter constructs. E, MFN2 expressions in VSMCs were detected by qRT‐PCR and Western blot with the co‐treatment of Hcy and DNMT1, DNMT3a and DNMT3b specific inhibitor (DC05, TFD and nanaomycin A). F, DNMT1 expression was detected by qRT‐PCR and Western blot after VSMCs were incubated with 100 μmol/L Hcy. G and H, Quantification of DNA methylation and the expression of MFN2 in VSMCs after overexpression or knock‐down of DNMT1. I, Representative immunofluorescence images of DNMT1 (red) and VSMCs marker (α‐SMA, green) in APOE^−/−^ mice, and nuclei were stained with DAPI (blue). Yellow indicated presence of both red and green staining, scale bar, 50mm. Quantification of the average DNMT1 intensity and co‐localization with α‐SMA was done, respectively. The experiment was performed in triplicate, and the representative images are shown. *P < 0.05, **P < 0.01, compared to the APOE^−/− ^+NC or Hcy+Ad‐GFP group, #*P* < 0.05, ##*P* < 0.01, compared to the Hcy+NC group

Transcriptional activity is key to determine gene expression levels in eukaryotic organisms, which is susceptible to various factors such as epigenetic modification including DNA methylation.^23^ To get insight into the underlying mechanism of MFN2 down‐regulation in VSMCs proliferation induced by Hcy, we analysed the sequence of the MFN2 promoter using the UCSC Human Genome Browser and NCBI gene bank (http://www.ncbi.nlm.nih. gov/pubmed/), and one CpG island between −885 and −783 at the proximal promoter of MFN2 relative to the TSS was found, meaning that MFN2 promoter has the potential to be methylated, which may alter its transcriptional activity (Figure 3A). Subsequently, several fragments of MFN2 5′‐flanking region (−1996/+1, −1043/+1, −885/+1, −783/+1 and −543/+1) were inserted into the firefly luciferase vector pGL3, and the luciferase activity assay revealed that the region from −885 to −783, which spans most of the CpG dinucleotide of MFN2 promoter region has the highest promoter activity (Figure 3B), indicating a possible regulatory element of MFN2 transcription in this region. To test this hypothesis, the methylation of the CpG islands was validated by MassARRAY Quantitative Methylation Analysis. As shown in Figure 3C, the amplified fragment with MassArray MFN2 primers contained 18 CpG sites, and the 14th CpG sites were not detected. The average of the methylation levels at all MFN2 loci obviously increased in VSMCs treated with Hcy, and which can be attenuated by azacytidine (AZC), an inhibitor of DNMTs activity. Moreover, proximal promoter region of MFN2 from −1043 to +1 was cloned and methylated by SssI, HhaI and HpaII to examine whether DNA methylation directly represses MFN2 promoter activity, among them, SssI methylases all 5′‐CpG‐3′sites (46 CpG sites), HhaI only methylases the CpG within the sequence 5′‐GCGC‐3′ (3 CpG sites), while HpaII methylases CpG within the sequence 5′‐CCGG‐3′ (5 CpG sites).^24^ Subsequently, proper methylation of them were confirmed by digestion with the restriction enzymes, including McrBC (methylation‐specific restriction enzyme), HhaI and HpaII, and the luciferase reporter assay showed that the promoter activity of the differentially methylated MFN2 proximal promoter region was markedly blocked after the methylation by SssI, HhaI or HpaII methylase, particular the SssI, showed the greatest repression of MFN2 promoter activity (Figure 3D). These results implied that MFN2 proximal promoter activity could be abrogated by DNA methylation.

DNA methylation in mammals is catalyzed by DNA methyltransferases (DNMTs) including maintenance (DNMT1) and de novo methyltransferases (DNMT3a, DNMT3b).^24^ Therefore, VSMCs were exposed to the specific inhibitor of these three DNMTs, DC‐05, TFD and nanaomycinA respectively when the cells were treated with Hcy, to make clear which enzyme was responsible for Hcy‐mediated transcriptional repression the MFN2, We found that treatment with DNMT1 inhibitor DC‐05 inhibits the expression of MFN2, while no significant differences of MFN2 expression were observed when treated with TFD and nanaomycinA respectively, implying that DNMT1 might be the key methyltransferase to regulate MFN2 expression in response to Hcy (Figure 3E). To validate whether DNMT1 participates in the transcriptional regulation of MFN2, qRT‐PCR and Western blot was subjected to detect DNMT1 expression and the results showed that DNMT1 expression was dramatically enhanced in VSMCs treated with Hcy (Figure 3F). Subsequently, Ad‐DNMT1 and DNMT1 siRNA were transfected into VSMCs to overexpress or silence DNMT1 expression (Supplementary Figure S2A,B). As expected, overexpression of DNMT1 in VSMCs significantly promoted methylation levels of MFN2 promoter and inhibited MFN2 expression in the presence of Hcy, and it was the opposite when DNMT1 was knocked down (Figure 3G,H). Given that the levels of DNA methyltransferases could reflect the DNA methylation to a certain extent, immunofluorescence staining of DNMT1 and α‐SMA showed that with the treatment of hyperhomocystinaemia, more double positive cells for α‐SMA and DNMT1 was found in APOE^−/−^ mice atherosclerotic plaque (Figure 3I). These data supported the hypothesis that DNMT1 is responsible for the hypermethylation of MFN2 promoter, which leads to suppression of MFN2 transcription in VSMCs treated with Hcy during the atherosclerotic plaque formation.

### 3.4 c‐Myc indirectly regulates the transcription of MFN2 in VSMCs

**Figure 4:**
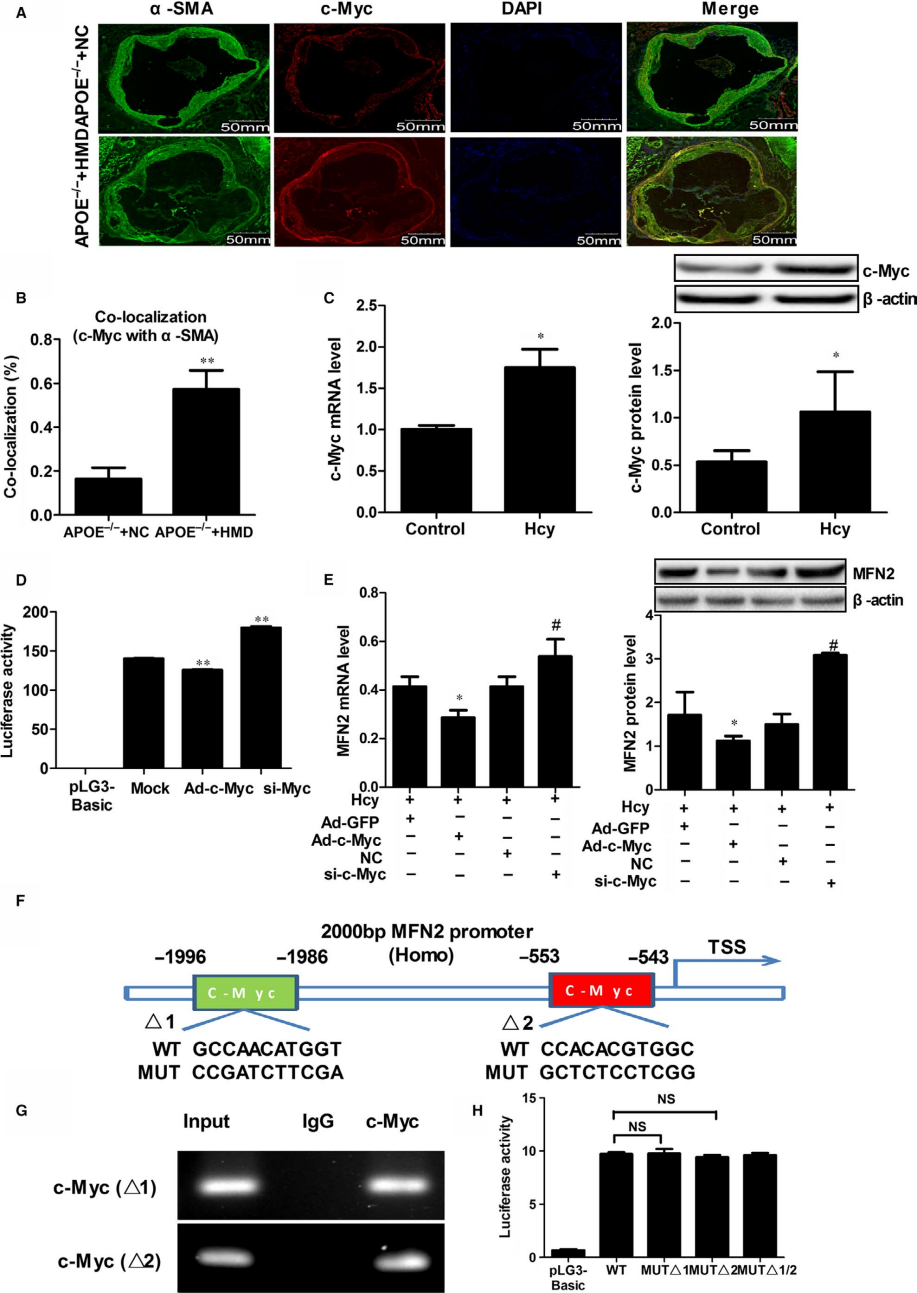
c‐Myc indirectly regulates MFN2 transcription in VSMCs. A and B, The co‐localization of c‐Myc (red) and α‐SMA (green) in the aortic root sections of APOE^−/−^ mice and nuclei were stained with DAPI (blue). Yellow indicated presence of both red and green staining, scale bar, 50mm. C, c‐Myc expression in VSMCs‐treated with 100 μmol/L Hcy were detected by qRT‐PCR and Western blot. D, Transcriptional activities of MFN2 proximal promoter in VSMCs was determined by luciferase reporter assay when overexpression or knock‐down c‐Myc, *P < 0.05, **P < 0.05 compared to the Mock group. E, The expression of MFN2 in VSMCs was detected by qRT‐PCR and Western blot when c‐Myc was overexpressed or knockdown in the presence of 100 μmol/L Hcy. F, The schematic representation of the two c‐Myc putative binding sites at MFN2 promoter (−2000 bp to TSS). G, ChIP assay were performed to examine the binding of c‐Myc to MFN2 promoter region in VSMCs treated with 100 μmol/L Hcy. According to the JASPAR database, we designed two primers containing the c‐Myc binding sites respectively. Chromatin‐bound DNA was immunoprecipitated with the anti‐c‐Myc antibody. IgG was used as a negative control. Thereafter, PCR was performed for the analysis of c‐Myc binding to the promoter of MFN2. H, Relative luciferase activities of MFN2 promoter were detected after solely or serially mutated c‐Myc binding sites at MFN2 promoter region by luciferase reporter assay, NS indicated no significant. *P < 0.05, **P < 0.01, compared to the APOE^−/−^+NC or Hcy+Ad‐GFP group. #*P* < 0.05, ##*P* < 0.01, compared to the Hcy+NC group

c‐Myc is a master regulator of cell proliferation and transformation, and it is implicated in numerous diseases.^25^ To illustrate whether c‐Myc is involved in the regulation of MFN2 transcription, we conducted double immunofluorescent staining of c‐Myc and α‐SMA in the aortic root sections of APOE^−/−^ mice, which showed that c‐Myc was distributed in both cytoplasm and nuclei of VSMCs, and the c‐Myc positive cells were much higher in APOE^−/−^ HMD group than that in APOE^−/−^ NC group (Figures 4A,B). Consistent with above, c‐Myc expression was dramatically increased in VSMCs treated with Hcy (Figure 4C). Subsequently, Ad‐c‐Myc or si‐c‐Myc were transfected into VSMCs. The luciferase reporter assay revealed that overexpression of c‐Myc significantly inhibits MFN2 promoter activity, which led to the low expression of MFN2 in VSMCs. Conversely, MFN2 promoter activity was strengthened in VSMCs when c‐Myc was knocked down (Figures 4D,E), suggesting that c‐Myc exerts a negative effect on MFN2 expression regulation. To further explore the potential mechanism on MFN2 transcription, the 2000 bp sequence of MFN2 promoter (NM_014874) was analysed by JASPAR database, and two putative c‐Myc binding sites “GCCAACATGGT” (−1996 to −1986) (∆1), “CCACACGTGGC” (−553 to −543) (∆2) were found at the MFN2 promoter (Figure 4F and Supplementary Figure S3). Subsequently, ChIP assays using an anti‐c‐Myc were applied to examine the binding of c‐Myc to MFN2 promoter region. The results demonstrated that c‐Myc binds to the MFN2 promoter at −1996‐−1986 and −553‐−543 (Figure 4G). Unexpectedly, both of the two c‐Myc binding sites were not located at the core regulation region of MFN2 promoter, and no evident changes in MFN2 promoter activity were observed by the mutation of these two binding sites (Figure 4H), indicating that the regulation of c‐Myc on MFN2 transcription in VSMCs is indirect.

### 3.5 Elevated binding of c‐Myc to DNMT1 promoter suppressed MFN2 transcription in VSMCs

**Figure 5:**
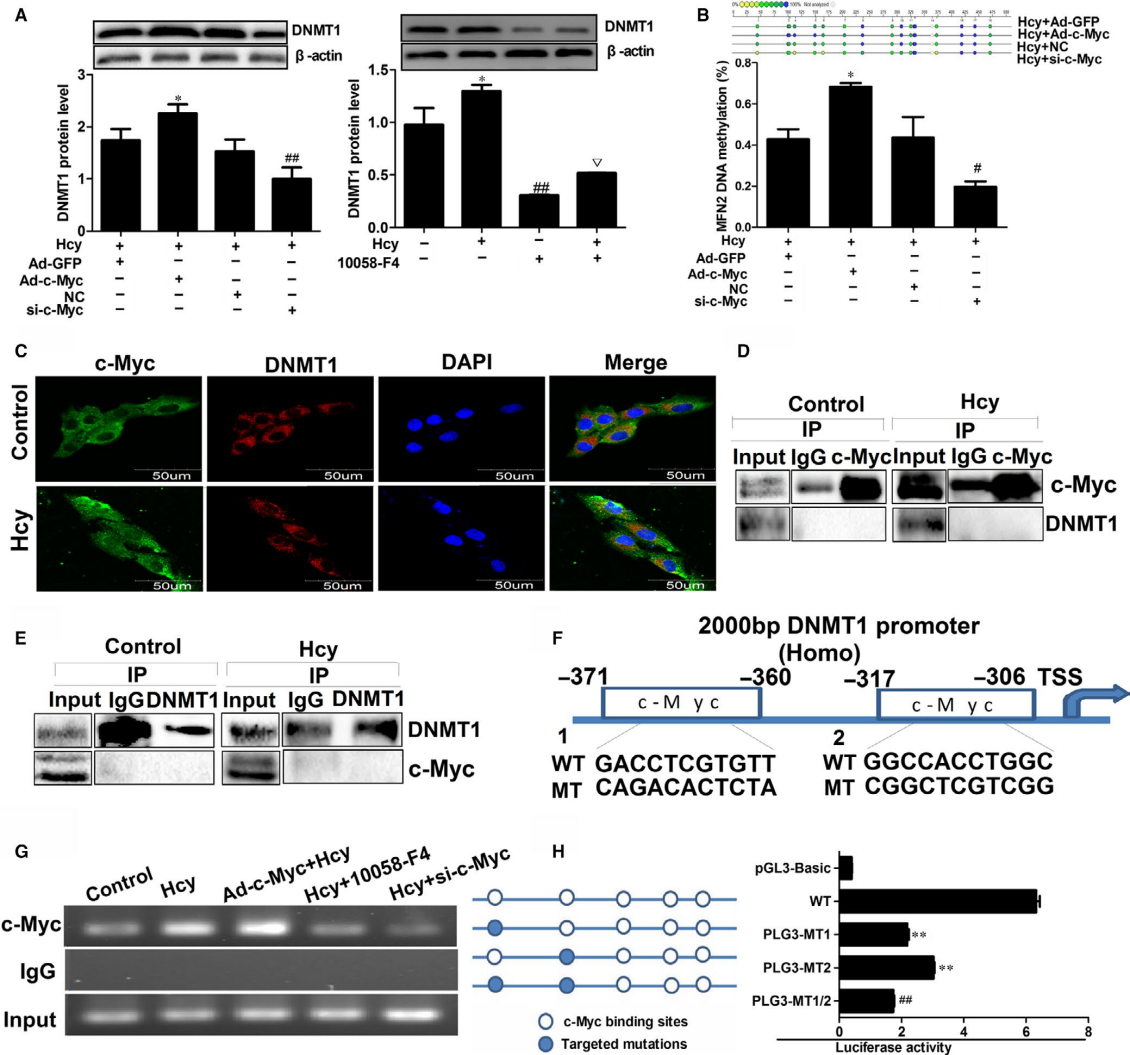
c‐Myc regulates MFN2 transcription through the increased binding to DNMT1 promoter in VSMCs. A, Protein expression of DNMT1 was detected by Western blot in VSMCs with the infection of Ad‐c‐Myc and transfection of c‐Myc RNA inference vector following with 100 µmol/L Hcy treatment. B, DNA methylation level of MFN2 promoter was analysed by Mass ARRAY Quantitative Methylation after the c‐Myc overexpression or knock‐down in VSMCs. C, Co‐location of c‐Myc (green) and DNMT1 (red) in VSMCs with the treatment of Hcy were observed by laser confocal microscopy. Nuclei were stained with DAPI (blue), scale bar, 50 μm. D and E, VSMCs was treated with the Hcy and then total cell lysates of VSMCs were immunoprecipitated with c‐Myc or DNMT1 antibodies or control IgG. Co‐IP samples were analysed by Western blot with the indicated antibodies. F, A schematic diagram of potential c‐Myc binding element (two possible binding sites) in the promoter region of the DNMT1 gene predicted by Jaspar database. G, ChIP assay was performed to assess the c‐Myc binding to MFN2 promoter region in VSMCs with the infection of Ad‐c‐Myc and transfection of RNA inference vector following with 100 μmol/L Hcy treatment. The ChIP‐enriched DNA fragments of MFN2 promoter using IgG and anti‐c‐Myc antibody were amplified by PCR. Total input (5%) was used as a positive control. H, Sequential deletion and substitution mutation analyses identified c‐Myc‐responsive regions in the DNMT1 proximal promoter region, the relative luciferase activities of DNMT1 promoter were detected after solely or serially mutated c‐Myc binding sites at DNMT1 promoter region by luciferase reporter assay. **P < 0.01, compared with the WT group, ##*P* < 0.01, compared with pLG3‐MT1 or pLG3‐MT2 group. *P < 0.05, **P < 0.01, compared to Hcy+Ad‐GFP group. #*P* < 0.05, ##*P* < 0.01, compared to the Hcy + NC group. ▽*P* < 0.05, compared to the 10058‐F4 group

To further explore the underlying mechanism of c‐Myc in the regulation of MFN2, Ad‐c‐Myc or a targeted siRNA were transfected into VSMCs. Interestingly, based on the treatment of Hcy, overexpression of c‐Myc enhanced the DNMT1 expression and MFN2 promoter methylation, while the tendency was opposite when c‐Myc expression was knocked down (Figure 5A,B). Additionally, similar effects were also observed in VSMCs after treatment with 10058‐F4, an inhibitor of c‐Myc (Figure 5A). Considering that c‐Myc was involved in the transcriptional regulation of genes in diverse ways, the subcellular locations of DNMT1 and c‐Myc in VSMCs were detected using laser confocal microscopy to explore the mechanism for c‐Myc–mediated MFN2 repression. The results showed that the distribution of DNMT1 was almost completely cytoplasm, while c‐Myc was distributed throughout both the nucleus and the cytoplasm as reported previously,^25^ and the co‐localization of these proteins in the cells remained unchanged under Hcy treatment (Figure 5C). We also employed protein co‐immunoprecipitation (Co‐IP) to examine the interactions between DNMT1 and c‐Myc. The whole‐cell extracts (WCE) of VSMCs treated with Hcy were immunoprecipitated with an anti‐c‐Myc antibody, and DNMT1 was not immunoprecipitated with c‐myc, meaning that Hcy does not promote the binding of DNMT1 and c‐Myc (Figure 5D). To confirm that, an anti‐DNMT1 antibody was used to perform a reverse protein Co‐IP experiment, and no co‐immunoprecipitation was also observed between c‐myc and DNMT1, and Hcy failed to promote binding of c‐myc to DNMT1 at the protein level. (Figure 5E). These results demonstrated that the effect of c‐Myc on DNMT1 was not attributed to the interaction between c‐Myc and DNMT1 at protein levels.

**Figure 6:**
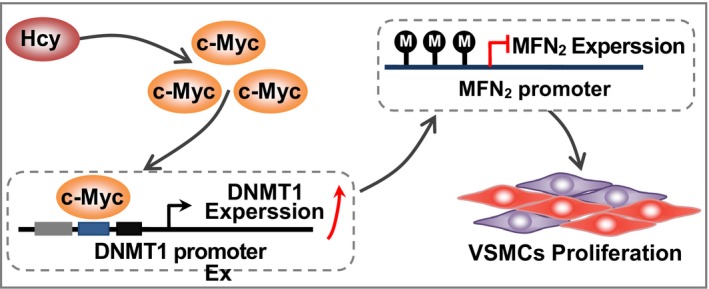
A proposed model of gene expression regulation in Hcy‐induced VSMCs proliferation. The hypermethylation of MFN2 mediated by DNMT1 promoter was involved in Hcy‐induced VSMCs proliferation in atherosclerosis, which attributed to increased binding of c‐Myc to DNMT1 promoter

Besides to interaction with other proteins, transcription factors can recognize DNA sequences in transcriptional regulation.^26^ In order to illustrate the underlying mechanism of c‐Myc in the regulation of DNMT1, the JASPER database was used to analyse the sequence of DNMT1 promoter, and two putative c‐Myc binding sites “GACCTCGTGTT” (−360‐−371), “GGCCACCTGGC” (−317‐−306) were observed (Figure 5F, Supplementary Figure S4). Subsequent ChIP assay showed Hcy significant promotes the c‐Myc binding to DNMT1 promoter region, and the binding is further increased when c‐Myc was overexpressed. Reciprocally, the binding of c‐Myc was inhibited with the low expression of c‐Myc via siRNA or 10058‐F4 (Figure 5G), which implied that the suppression of c‐Myc on MFN2 transcription in VSMCs treated with Hcy may be ascribed to the c‐Myc binding to DNMT1 promoter. To support our hypothesis, we generated wild‐type and two mutated putative c‐Myc binding sites, and transcriptional activity of DNMT1 promoter was assessed by luciferase reporter assay. The results showed that DNMT1 promoter activity could be attenuated when mutated in either of the two binding sites, and mutation of both binding sites will lead to the further reduction of DNMT1 promoter activity (Figure 5H). Collectively, these data suggested that the increased binding of c‐Myc to DNMT1 promoter region is responsible for the negative regulation of c‐Myc on MFN2 transcription in VSMCs proliferation induced by Hcy (Figure 6).

## 4 DISCUSSION

Over the past decade, a wealth of studies showed that abnormal VSMCs proliferation is a key event in the development of atherosclerotic plaque.^27^ Given its importance, a detailed understanding of the molecular mechanism factors on aberrant VSMCs proliferation during atherosclerotic plaque formation is imperative. Here we presented evidence about the critical role and molecular mechanism of MFN2 in regulating VSMCs proliferation during the formation of atherosclerotic plaque. Our data demonstrated that the binding of c‐Myc to DNMT1 promoter facilitates MFN2 hypermethylation and suppresses MFN2 transcription activity. This finding provides a new insight into Hcy‐associated VSMCs proliferation in the process of atherosclerotic plaque formation.

Atherosclerosis is a complex disease which begins with eccentric thickening of the intima, which is predominantly composed of VSMCs, mesenchymal intimal cells and inflammatory cells.^2^ In recent years, VSMCs proliferation has been considered as an important pathological factor in atherosclerosis.^2^ Hcy is an independent risk factor of atherosclerosis.^28^ Evidences revealed a marked stimulatory effect of Hcy on VSMC proliferation.^13^ It was well accepted that the transition of cell cycle from G0/G1 phase to S phase is one of the important characteristics of cell proliferation, which is accompanied by a great increase in DNA synthesis and abnormal expression of cell cycle proteins.^29^, ^30^ p27 can block the cell cycle in G0/G1 phase by negatively regulation of cyclin/CDK complexes, and PCNA is a well‐known molecular marker for cell proliferation because of the role of replication in S phase, which could coordinate with p27 in the regulation of cell cycle.^31^, ^32^ In this study, we found that the proliferation of VSMCs is enhanced in the atherosclerotic plaque of APOE^−/− ^mice with hyperhomocystinaemia, as evidenced by the increased PCNA expression and decreased p27 expression. Meantime, similar result was also observed in VSMCs treated with Hcy in vitro. Considering that the classification of hyperhomocysteinaemia in human is defined with respect to serum concentration as follows: moderate (15‐30 µmol/L), intermediate (30‐100 µmol/L), severe (>100 µmol/L),^29^ a wide range of Hcy concentrations (50, 100, 200, 500 μmol/L) that are similar as that in patients with hyperhomocysteinaemia was used to stimulate VSMCs. Consequently, more proportion of cells advancing into S phase from G0/G1 phase was observed in response to Hcy at 100 μmol/L. We have confirmed that Hcy apparently promoted VSMCs proliferation with 100 μmol/L Hcy most potential. Considering that VSMCs proliferation is inhibited with the concentration of Hcy increases, especially greater than 100 μmol/L, implying that there may be other regulatory mechanisms. Therefore, we used 100 μmol/L Hcy in further experiments in order to well demonstrate the role of Hcy in VSMCs proliferation. 100 μmol/L Hcy was also used in other studies^33^, ^34^ to explore the effects of Hcy on VSMCs proliferation and human aortic smooth muscle cells (HASMC) mitochondrial dysfunction and it was showed that Hcy significantly promotes VSMCs proliferation, which further confirmed our results.

The pathogenesis of atherosclerosis involves changes in the expression and function of a series of genes, and VSMCs proliferation is precisely regulated by various proliferation related genes, particularly the mitogenic genes. MFN2 is a newly discovered cell proliferation inhibitor that localize in the mitochondrial outer membrane and it plays an essential role in mitochondrial fusion, mitochondrial morphology in mammalian cells, yeasts, and flies.^35^, ^36^ It was reported that MicroRNA‐497 promotes cardiomyocytes proliferation through the down‐regulation of MFN2 in a mouse model of myocardial ischaemia‐reperfusion injury.^37^ Our results revealed that expression of MFN2 is down‐regulated both in atherosclerotic plaque of APOE^−/−^ mice with high‐methionine diet and Hcy‐treated VSMCs, and overexpression of MFN2 inhibits of VSMCs proliferation by the arrest at G0/G1 phase. Similar results were also found in cancer cells, where MFN2 exert an anti‐proliferative effect by inducing cell cycle arrest in the G0/G1 phase via inhibition of Ras‐Raf‐ERK1/2 signalling pathway.^38^ These previous observations and this study suggest that Hcy can promote proliferation of VSMCs during the formation of atherosclerotic plaque, which is at least partially dependent on the down‐regulation of MFN2 expression.

Given the fact that the proximal promoter and 5′‐untranslated region of MFN2 is enriched with CpG sites, we found that the methylation levels are significantly elevated in response to Hcy, which is accompanied by the inhibition of MFN2 transcription. DNA methylation is catalyzed by DNMTs including DNMT1, DNMT3a and DNMT3b.^24^, ^39^ Here, we found that MFN2 transcription was modulated by DNMT1, as exhibited by the notably up‐regulated MFN2 expression when VSMCs were exposed to the specific DNMT1 inhibitor DC_05. This observation is consistent with the previous report that promoter hypermethylation is associated with transcriptional suppression,^40^ suggesting that DNA methylation plays an important role in VSMCs proliferation during atherosclerotic plaque formation. What regulates DNMT1 to methylate the MFN2 promoter and inhibits its expression in a cell type‐specific manner should be further investigated. The current prevailing explanation for Hcy‐induced hypermethylation of MFN2 is that DNA methylation modification needs S‐adenosylmethionine (SAM) to be as the methyl donor. After transmethylation reaction, SAM transfers to S‐adenosylhomocysteine (SAH) that undergoes hydrolysis to form Hcy. Hcy can be remethylated to methionine and then methionine is converted to SAM.^41^ The methionine cycle would be promoted with the increased levels of Hcy, which lead to an increased generation of SAM and alter the status of DNA methylation of some specific genes that involved in the pathogenesis of atherosclerosis. In addition, the levels of DNA methylation will influence the status of chromatin structure to be dense and impede gene transcription. Meanwhile, cytosine methylated will alter the spatial structure of DNA molecules, which directly interferes the binding of specific transcription factors to the promoter of the targeted gene, by which it suppressed the target gene transcription activity. Nevertheless, how can Hcy suppress MFN2 transcriptional activity was still not elucidated, so this explanation for hypermethylation is still plausible.

Previous studies have discussed various ways to target transcription factors in disease models: by modulating their expression or degradation, by targeting the transcription factor itself to prevent its DNA binding either through a binding pocket or at the DNA‐interacting site and so on.^42^ c‐Myc is a highly pleiotropic transcription factor that can regulate cell cycle progression, proliferation and metabolism.^43^ Recently, c‐Myc has been considered as a key factor in the transcriptional response to induce the transition of hepatocytes from G0/G1 to the S phase.^44^ In accordance with the previous findings,^45^ our study showed that c‐Myc expression was up‐regulated in VSMCs treated with Hcy and atherosclerotic plaque in APOE^−/−^ mice which were fed with high‐methionine diet. Additionally, the regulation of c‐Myc on MFN2 expression was indirect, as showed by the increased binding of c‐Myc at DNMT1 promoter in response to Hcy lead to MFN2 suppression, while no obvious transcriptional activities of MFN2 was observed when mutated the c‐Myc binding sites, indicating that c‐Myc is involved in the inhibition of MFN2 transcription activities through DNMT1 in VSMCs proliferation treated with Hcy. A previous study provided striking molecular evidence that besides to recognize the binding sites located at promoter of the targeted genes, another way for transcription factors to exert its effect is dependent on the interaction with other proteins.^26^ Interestingly, our data demonstrated no significant interaction between c‐Myc and DNMT1 protein in VSMCs treated by Hcy, on the contrary, the enrichment of c‐Myc on DNMT1 promoter region was increased, suggesting that the enrichment of c‐Myc at DNMT1 promoter played a dominant role in the suppression of MFN2 transcriptional activity in VSMCs proliferation induced by Hcy. The reasons may be as following: First, c‐Myc has been shown to recruit DNMTs such as DNMT1 to repress the MFN2 gene expression. It raises the question that c‐Myc might be involved in the DNA methylation complex, particularly when MFN2 is a target. Meantime, aberrant expression of DNA methyl transferase has been detected in VSMCs that could affect the status of DNA methylation. Second, MFN2 has special infrastructure of promoter region and the transactivation domain was the region (−885/−783), where spans most of the CpG island that has the potential to be methylated and was essential to alter transcriptional activity. Notably, c‐Myc binding sites on MFN2 promoter were not at transactivation domain, where it has a slight effect on transcriptional activity of MFN2. Last but not least, Hcy is a sulphur‐containing amino acid, which could provide methyl for DNA from SAM to SAH, the activity of endogenous DNMTs was increased with the increase in Hcy concentration,^46^ which lead to the hypermethylation of MFN2. Additionally, Hcy promoted c‐Myc expression in VSMCs and facilitated the enrichment of c‐Myc at DNMT1 promoter contributed to the up‐regulation of DNMT1, and further resulting in a hypermethylation of MFN2 promoter and the MFN2 transcriptional activity was suppressed.

In conclusion, our study suggested that the increased binding of c‐Myc to DNMT1 promoter mediated the hypermethylation of MFN2 promoter leading to the MFN2 transcriptional activity suppression, which contributed to the abnormal proliferation of VSMCs in the process of atherosclerotic plaque formation. These findings identified a new regulatory mechanism of MFN2 transcription and shed insight into deeper understanding of aberrant VSMCs proliferation in atherosclerotic plaque. Meantime, the interrelations study of c‐Myc and DNMT1 on the regulatory mechanisms of MFN2 will provide a therapeutic target for Hcy‐induced cardiovascular diseases.

## CONFLICT OF INTEREST

The authors declare that there are no conflicts of interest.

## AUTHORS’ CONTRIBUTIONS

LX, WG, HH, YH and GL conceived and performed all the experiments in the Lab; PM, LX and ND researched the data contributed to the statistical analysis and discussion; SM optimized some key technological steps; AF aided with the experimental arrangements and technological works; YJ contributed to the discussion and reviewed the manuscript analysed the results. All authors reviewed the manuscript.
