# Supplementary figures and images for: Aberrant MFN2 transcription facilitates homocysteine‐induced VSMCs proliferation via the increased binding of c‐Myc to DNMT1 in atherosclerosis
Source: J Cell Mol Med. 2019 May 18;23(7):4611–26. doi: 10.1111/jcmm.14341 (PMC6584594; doi:10.1111/jcmm.14341)

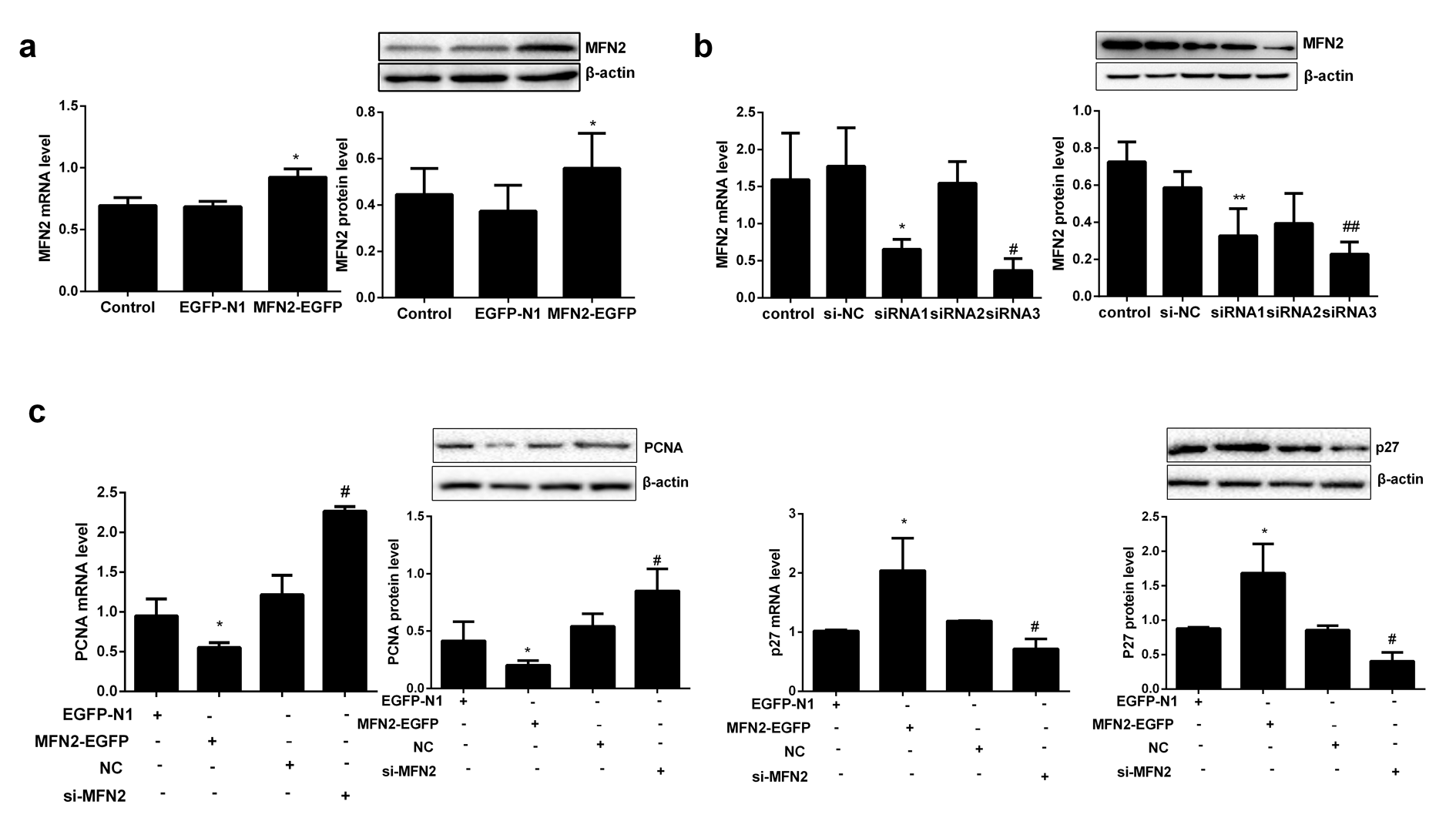

Supplement: Supplementary file 1 [file JCMM-23-4611-s001.tif]

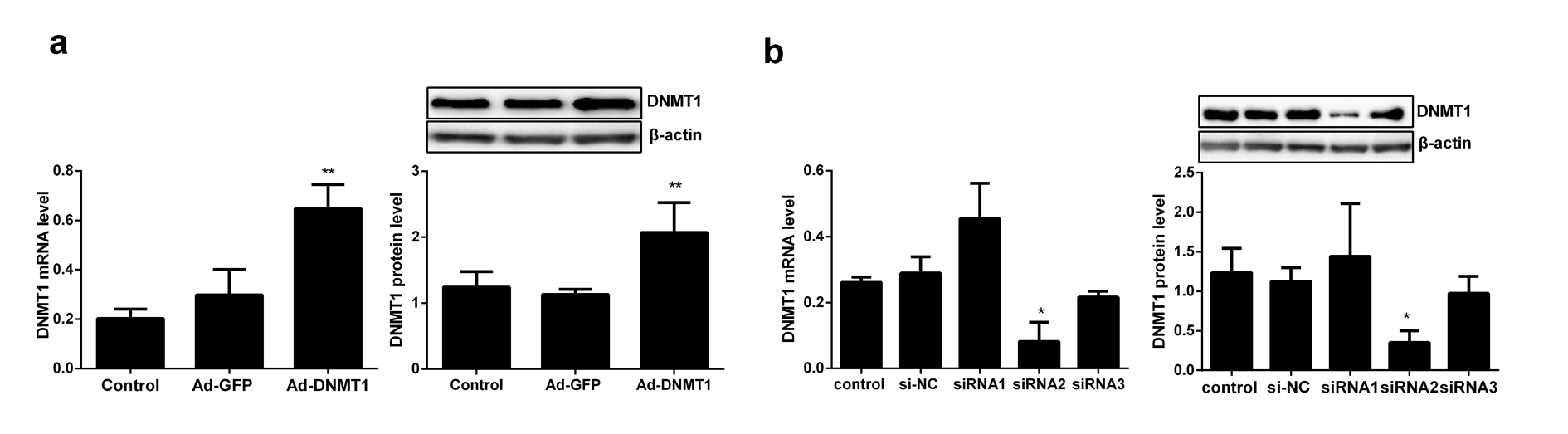

Supplement: Supplementary file 2 [file JCMM-23-4611-s002.tif]
